# DNA-Redox Cation Interaction Improves the Sensitivity of an Electrochemical Immunosensor for Protein Detection

**DOI:** 10.3390/s150820543

**Published:** 2015-08-20

**Authors:** Ping Li, Bixia Ge, Lily M.-L. Ou, Zhihui Yao, Hua-Zhong Yu

**Affiliations:** 1 Department of Chemistry, Simon Fraser University, 8888 University Drive, Burnaby, BC V5A 1S6, Canada; E-Mails: pingl@sfu.ca (P.L.); lilyo@sfu.ca (L.M.-L.O.); 2 Biogate Laboratories Ltd., 110-4238 Lozells Avenue, Burnaby, BC V5A 0C4, Canada; E-Mails: bge@sfu.ca (B.G.); yaozhihui@hotmail.com (Z.Y.)

**Keywords:** electrochemical immunoassay, redox labeling, signal enhancement, DNA-cation interaction, hCG

## Abstract

A simple DNA-redox cation interaction enhancement strategy has been developed to improve the sensitivity of electrochemical immunosensors for protein detection. Instead of labeling with fluorophores or redox-active groups, the detection antibodies were tethered with DNA single strands. Based on the electrostatic interaction between redox cations ([Ru(NH_3_)_6_]^3+^) and negatively charged DNA backbone, enhanced electrochemical signals were obtained. Human chorionic gonadotropin (hCG) detection has been performed as a trial analysis. A linear response range up to the concentration of 25 mIU/mL and a detection limit of 1.25 mIU/mL have been achieved, both are comparable with the ultrasensitive enzyme-linked immunosorbent assay (ELISA) tests. The method also shows great selectivity towards hCG over other hormones such as thyroid stimulating hormone (TSH) and follicle stimulating hormone (FSH). By and large, our approach bears the merits of cost effectiveness and simplicity of instrumentation in comparison with conventional optical detection methods.

## 1. Introduction

Immunoassays are among the most active research topics in several paramount fields, such as medical diagnosis [1], drug discovery [2], food safety testing [3], and environmental monitoring [4]. By utilizing the specific recognition of antibody/antigen interactions, the immunoassay test provides an extraordinarily selective and sensitive way to quantitate target analytes of interest [5]. Enzyme-linked immunosorbent assay (ELISA), one of the most widely used and reliable techniques for quantitative protein detection, was invented half a century ago [6]. This technology allows one to detect and quantitate molecular analytes such as antibodies, peptides, and hormones by binding antigens onto solid surfaces and recognizing them with specific antibodies that are tethered with enzymes for signal enhancement [7]. Horseradish peroxidase (HRP) is commonly used in ELISA to catalyze the reaction between 3,3ʹ,5,5ʹ-tetramethylbenzidine (TMB) and H_2_O_2_; the blue colored product is quantitated by UV/Vis spectrophotometry [8].

Recently, immunoassays based on various signaling techniques, such as radioimmunoassay [9], colorimetric assay [10], fluorescence immunoassay [11], and electrochemical assay [12], have been developed to merge the benefits of different approaches [13]. Among those, electrochemical immunosensors exhibit the merits of cost effectiveness, good portability, and excellent sensitivity [14]. Signal amplification for electrochemical immunosensors is crucial for the purpose of early detection of disease-related protein biomarkers when present in patient samples at ultralow levels [15]. Several strategies for signal enhancement have been reported, among which amplification of the throughput with enzymes is most notable [16,17]. Yang and coworkers reported an alkaline phosphatase (ALP) enzyme-labeled immunosensor for the detection of cardiac troponin I in human serum based on the catalytic generation of L-ascorbic acid (AA) and its redox recycling by tris(2-carboxyethyl)phosphine (TCEP) [18]. However, the protein enzyme labelling is not easily achieved, and such modification may influence the specific binding epitopes and the biological activity of the enzyme [19]. In the meantime, redox center-tethered immunosensors have been explored; Wang *et al.* reported a graphene/gold nanoparticle conjugated immunosensor using ferrocene as label for human IgG detection [20]. 

Instead of covalent labeling an antibody with a single redox molecule, herein we explore a conceptually different signal enhancement method for protein detection with electrochemical immunosensors. Inspired by Guo and co-workers’ strategy of using dye molecule/double-stranded DNA conjugates as labels for ultrasensitive fluorescence detection [21], we hope to achieve enhanced electrochemical signals from surface-bound redox cations (e.g., [Ru(NH_3_)_6_]^3+^) upon labeling the detecting antibody with DNA strands in a standard sandwich-format immunoassay. Multiply charged redox cations (e.g., [Ru(NH_3_)_6_]^3+^) are well known to strongly bind to a negatively charged DNA phosphate backbone by electrostatic interaction [22]; upon saturation the surface density of [Ru(NH_3_)_6_]^3+^ can be calculated from the integrated charge of the voltammetric measurements, which in turn provides a quantitative measure of the antigens to be detected in the immunoassays.

In our experiments, human chorionic gonadotropin (hCG) detection has been adopted as a model system for proof-of-concept. hCG is a glycoprotein hormone produced by conceptus during pregnancy. It is a heterodimer consisting of two subunits, α and β, which attach to each other non-covalently [23]. In human fluids, hCG is present in many forms such as hCGα subunit, hCGβ subunit, intact hCG, and hCG β fragment. Beyond the early pregnancy tests (done qualitatively), the quantitative determination of the hCG amount in urine can be used to monitor abnormal pregnancy conditions and to diagnose other complications such as testicular cancer and gestational trophoblastic diseases [24,25,26]. The conventional quantitative hCG detection method is ELISA, which uses a 96-well microtiter plate for preparing the assay and a spectrophotometer-type plate reader to read the signal. It must be performed in a biomedical laboratory setting by well-trained technicians [27].

## 2. Experimental Section

### 2.1. Reagents and Materials

The human chorionic gonadotropin (hCG) standard samples (100 mIU·mL^−1^), anti-hCGα monoclonal antibody (Mab), anti-hCGβ Mab, follicle stimulating hormone (FSH), and thyroid stimulating hormone (TSH) standard samples were provided by Biogate Laboratories Ltd. (Burnaby, BC, Canada). The 27-mer synthetic oligonucleotide, 5′-biotin-GTC CGT GGT AGG GCA GGT TGG GGT GAC-3ʹ, 42-mer synthetic oligonucleotide, 5ʹ-biotin-ATC TAC GAA TTC ATC AGG GCT AAA GAG TGC AGA GTT ACT TAG-3ʹ, and the complementary strand 5ʹ-CTA AGT AAC TCT GCA CTC TTT AGC CCT GAT GAA TTC GTA GAT-3ʹ were purchased from Integrated DNA Technologies, Inc. (Coralville, IA, USA). Gold substrates (regular glass slides coated first with 5-nm Cr, then with 100-nm Au) were purchased from Evaporated Metal Films (EMF) Inc. (Ithaca, NY, USA). The biotin labeling kit was purchased from Dojindo Molecular Technologies, Inc. (Rockville, MD, USA). *N*-Hydroxysuccinimide (NHS), 1-ethyl-3-(3-dimethylaminopropyl) carbodiimide hydrochloride (EDC), *N*-morpholinoethane sulfonic acid (MES), 6-mercapto-1-hexanol (MCH), 6-mercaptohexanoic acid (MHA), hexaammineruthenium (III) chloride (98%), and Tween-20 were purchased form Sigma-Aldrich (Milwaukee, WI, USA). MPEG3-NH_2_ (C_7_H_17_NO_3_) was purchased from ChemPep Inc. (Wellington, FL, USA). Deionized water (>18.3 MΩ·cm) was produced in a Barnstead EasyPure UV/UF compact water system (Dubuque, IA, USA).

The buffer solutions had the following compositions. Activation buffer: 0.1 M *N*-morpholinoethane sulfonic acid (MES), pH 5.8; immobilization buffer: 100 mM phosphate buffer, 150 mM NaCl, 5% glycerol, pH 7.4; washing buffer: 100 mM phosphate buffer, 150 mM NaCl, 0.1% gelatin, 0.05% Tween 20, 5% glycerol and 2 mM NaN_3_ at pH 7.4.

### 2.2. Substrate Modification and Immunosensor Preparation

Small pieces of gold slides (0.7 × 2.5 cm) were cleaned by immersion in a piranha cleaning solution (3:1 mixture of 98% H_2_SO_4_ and 30% H_2_O_2_) for 5 min at 90 °C, followed by a rinse with copious amounts of deionized water. *CAUTION: Piranha solution reacts violently with organic solvents and must be handled with extreme caution.* The gold chips were then dried with N_2_. A 15-μL drop of the binary solution of 5 mM MHA and 10 mM MCH was spread over the freshly cleaned gold substrates and incubated for one hour at 100% humidity. After the modification, the gold slides were rinsed with deionized water, followed by adding a 15-μL drop of the activation buffer (containing 100 mM EDC and 25 mM NHS) and incubating at room temperature for 3 h. 

For the preparation of the hCG sandwich assay a 15-μL drop of the capture antibody, anti-hCGα Mab (50 μg·mL^−1^) in the immobilization buffer was spread over each gold chip and kept overnight at 100% humidity. Unbound anti-hCGα Mab was washed away later with immobilization buffer. The unreacted carboxylic acid groups on the surface were blocked by reaction with 0.1 M MPEG3-NH_2_ (15 μL on each gold chip) for an hour. The hCG samples in the immobilization buffer were then spread on the modified gold slides (15 μL on each slide) and incubated for an hour. Biotin-labeled anti-hCGβ Mab (0.1 μg·mL^−1^) in washing buffer was then added onto each gold chip and kept for one hour. The gold chips were rinsed with washing buffer, followed by adding a drop of 15 μL streptavidin (25 μg·mL^−1^) in washing buffer and incubation for one hour. A 15-μL drop of 3 μM biotin-labeled DNA strands in TE buffer (10 mM Tris, 1 mM EDTA, pH 8) was spread over each gold chip and incubated for one hour after washing away the unreacted streptavidin.

For the double-stranded DNA labeling, the biotin-labelled DNA single strand was hybridized with its complementary strand in hybridization buffer (10 mM Tris, 150 mM NaCl, 3 mM MgCl_2_) by incubating at 80 °C for 5 min, followed by slow cooling to room temperature over a period of 60 min to form biotin-labelled double-stranded DNA. 

### 2.3. Electrochemical Measurements

Cyclic voltammetry (CV) measurements were performed with a CHI 660D electrochemical workstation (CH Instruments Inc., Austin, TX, USA) using a three-electrode single chamber cell made of Plexiglas V-series acrylic resin. The working electrode (antibody-antigen modified gold slide) was pressed against an O-ring seal (with an exposed area of 0.126 cm^2^) at the side of the cell. A platinum wire and an Ag | AgCl | 3 M NaCl electrode were used as counter and reference electrode, respectively. 

All electrochemical measurements were performed with 5.0 μM [Ru(NH_3_)_6_]Cl_3_ in 10 mM Tris-HCl buffer at pH 7.4 under ambient conditions (21–23 °C). The supporting electrolyte was deoxygenated by bubbling argon for at least 15 min prior to the measurement.

## 3. Results and Discussion

### 3.1. DNA-Redox Cation Interaction Enhanced Electrochemical Immunoassay for hCG Detection

**Figure 1:**
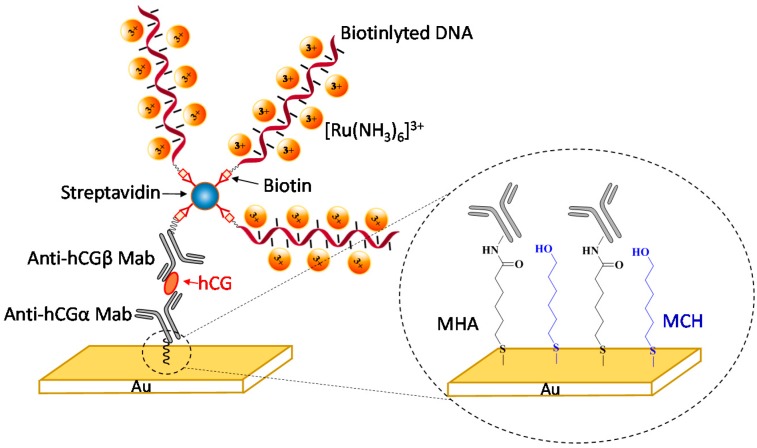
Design of a sandwich-format electrochemical immunoassay using hCG as a trial analyte. Right Inset: anti-hCGα Mab were attached to the gold surface by amide coupling with carboxylic acid groups on a mixed MHA/MCH monolayer.

As shown in Figure 1, a sandwich-format immunoassay was constructed on a gold electrode for the detection of hCG. Two different monoclonal antibodies were employed: the capture antibody anti-hCGα Mab and the detection antibody anti-hCGβ Mab. As mentioned above, hCG is a heterodimer that consists of an hCGα subunit and an hCGβ subunit. Due to the presence of several epitope binding sites on an hCG dimer, the anti-hCGα Mab can bind both hCGα subunit and hCG dimer; anti-hCGβ Mab can bind hCGβ subunit, hCG β-core fragment, and hCG dimer [28]. Therefore, this capture/detection antibody pair can recognize not only the intact hCG hormone but also the core-fragments, which provides a means of quantitating the total hCG level in a sample of interest.

A mixed self-assembled monolayer (SAM) of 6-mercaptohexanoic acid (MHA) and 6-mercapto-1-hexanol (MCH) on gold provides carboxylic acid groups to immobilize antibodies; the inert alkyl chains serve as “spacers” to reduce the surface density of the active sites. To facilitate the immobilization of capture antibodies, the surface was treated with EDC and NHS to activate the carboxylic acid groups. In order to reduce the potential nonspecific adsorption, the unreacted carboxylic acid groups were blocked by reaction with MPEG3-NH_2_ (C_7_H_17_NO_3_). Unlike a standard ELISA assay, the detection antibody was labeled with a biotin group for binding streptavidin that was subsequently tethered with DNA strands. As depicted in Figure 1, multiple DNA strands were attached to the sandwich structure by binding the streptavidin on the surface. The fact that each streptavidin has four biotin-binding sites, we expected that up to three biotinylated DNA strands would be attached to a single detection antibody. 

**Figure 2:**
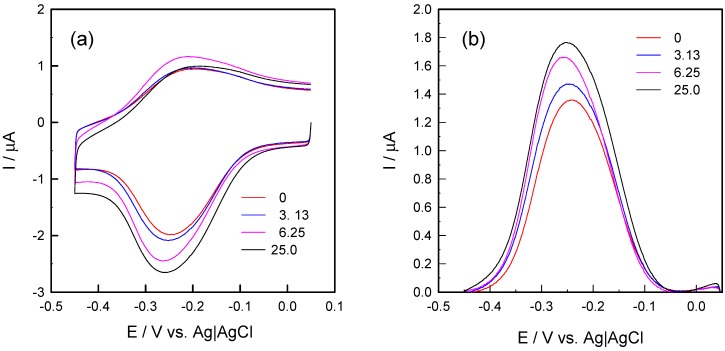
(**a**) Cyclic voltammograms of 5.0 μM [Ru(NH_3_)_6_]^3+^ on sandwich-format immunoassay-modified gold electrodes in 10 mM Tris buffer (pH 7.4) upon incubation with hCG standard solutions; (**b**) Normalized cathodic peaks at four representative concentrations of hCG: 0, 3.13, 6.25, 25.0 mIU/mL.

Figure 2 shows the CV responses of the sensor electrodes that were incubated with hCG standard samples of different concentrations in the presence of 5.0 μM [Ru(NH_3_)_6_]^3+^. In retrospect, the application of multiply charged redox cations (e.g., [Ru(NH_3_)_6_]^3+^) for the quantitation of DNA probes on electrode surfaces has been developed as a routine practice [29,30]. The validity of this method relies on the following assumptions: (1) the electrostatic interaction is the only binding force between redox cations and DNA strands; (2) the determination of the amount of trapped redox cations is accurate; and (3) other cations such as Na^+^ and K^+^ adsorbed onto DNA phosphate backbones can be completely replaced by [Ru(NH_3_)_6_]^3+^, which means the charge compensation of the DNA phosphate backbones is exclusively provided by redox cations [26]. Because of the high binding affinity to the DNA monolayer, a low concentration of [Ru(NH_3_)_6_]^3+^ (<5.0 μM) is suitable for ensuring that the saturation of the DNA-modified surface with redox-active cations produces negligible background current (of the diffused species) [31,32]. 

As shown in Figure 2b the cathodic peak area (*i.e.*, reduction of [Ru(NH_3_)_6_]^3+^ to [Ru(NH_3_)_6_]^2+^) increased monotonically with increasing the hCG concentration. The cathodic peak appeared at −0.25 V *vs.* Ag|AgCl in the absence of hCG and exhibited a gradual shift toward the negative direction. Such a formal potential shift shows the rather strong electrostatic interaction between DNA strands and [Ru(NH_3_)_6_]^3+^, particularly the different binding affinities of the oxidized ([Ru(NH_3_)_6_]^3+^) and the reduced forms ([Ru(NH_3_)_6_]^2+^) [33,34]:

(1) $$\Delta E{{}^{\circ}}^{'}=-\frac{RT}{nF}1\text{n}\left(\frac{K_{\text{Ox}}}{K_{\text{Red}}}\right)$$

The gradual increase in the cathodic peak currents confirms the accumulation of redox cations in the DNA layer. Accompanied by higher concentrations of hCG, the formal potential shifted slightly to a more negative value, showing the differences in binding strength between [Ru(NH_3_)_6_]^3+^ and DNA with increased surface densities. The non-zero background signal and the cathodic peak with no analyte added indicate the binding of [Ru(NH_3_)_6_]^3+^ to the capture antibodies or direct interaction between the capture and detection antibodies. Another possibility is electrostatic interaction between [Ru(NH_3_)_6_]^3+^ and unreacted carboxylic acid groups in the mixed MHA/MCH monolayer. 

**Figure 3:**
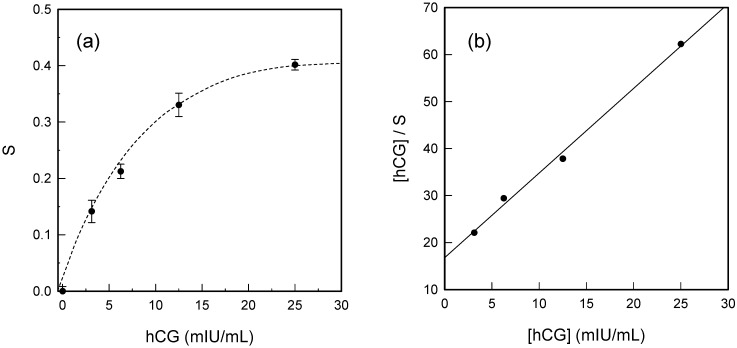
(**a**) Relative signal increase (*S*) as a function of concentration of hCG; (**b**) Linearized adsorption isotherm of hCG binding to anti-hCGα monoclonal antibodies on the gold electrode based on the Langmuir model. The solid line is the best fit to the experimental data from which the dissociation constant *K_d_* was determined.

To quantitatively evaluate the detection limit and the response range, the relative increase in the integrated charge of the reduction peak has been determined. In particular, the dependence of the relative signal increase (*S*) obtained by integration of the reduction peak of [Ru(NH_3_)_6_]^3+^ in the CVs before (*Q*_0_) and after (*Q*) incubation with hCG, *S* = (*Q − Q*_0_)/*Q*_0_, with the concentration of hCG was plotted. As shown in Figure 3a, a clear increase of the sensor signal was observed with increasing the hCG concentration. The sensor signal reaches saturation when the concentration of hCG increases to 25 mIU/mL, in which case we can determine the saturated sensor signal, *S*_sat_ = (*Q*_max_ − *Q*_0_)/*Q*_0_. We note that for the entire concentration range, the signal is not linearly related to the concentration. However, at low concentrations (up to 12.5 mIU/mL), the relative signal increase may be considered proportional to the hCG concentration within the experimental uncertainties. The detection limit of this sandwich-format immunosensor was estimated to be 1.25 mIU/mL of hCG, which is three times as the standard deviation of the signal. It is noteworthy that this detection limit is comparable to that of the conventional ELISA test [35].

For a better understanding of the antigen-antibody interaction on a surface, the adsorption isotherm of hCG binding to anti-hCGα monoclonal antibodies has been further analyzed. Assuming the binding process meets the requirements of Langmuir isotherms, the dissociation constant (*K*_d_) can be calculated from:

(2) $$\text{Mab}{|}_{\text{gold}}+\text{hCG}⇌\text{Mab}•\text{hCG}{|}_{\text{gold}}$$

(3) $$K_{d}=\frac{\left[\text{Mab}\right]\left[\text{hCG}\right]}{\left[\text{Mab}•\text{hCG}\right]}$$

where [Mab] and [Mab•hCG] are the surface concentrations of unbounded and bounded anti-hCGα Mabs upon reaching binding equilibrium. If we define θ, the fractional occupancy, as the ratio between bounded Mab to the total surface concentration of Mab, [Mab•hCG]/([Mab] + [Mab•hCG]), the above equation can be rearranged as:

(4) $$\text{θ}=\frac{\left[\text{hCG}\right]}{K_{d}+\left[\text{hCG}\right]}$$

Because the amount of [Mab•hCG] adsorbed onto the surface is proportional to the electrochemical signal *S* defined above, θ can be obtained from:

(5) $$\text{θ}=\frac{S}{S_{sat}}$$

We then have:

(6) $$\frac{S}{S_{sat}}=\frac{\left[\text{hCG}\right]}{K_{d}+\left[\text{hCG}\right]}$$

However, in a sandwich assay both capture antibody and detection antibody affect the binding behavior of the antigen, herein we only consider the first-step binding by assuming similar affinities of the two antibodies to hCG. Therefore, we applied the following equation to estimate the *K*_d_ value [36,37,38]:

(7) $$\frac{\left[\text{hCG}\right]}{S}\text{=}\frac{\left[\text{hCG}\right]}{S_{sat}}\text{+}\frac{K_{d}}{S_{sat}}$$

Figure 3b shows that [hCG]/S is indeed proportional to [hCG], indicating that the binding process does follow a Langmuir isotherm model. From the best linear fit, *K_d_* was calculated to be (2.2 ± 0.2) × 10^−11^ M, which is in general agreement with the value determined by Englebienne *et al.* [39]. Beside the determination of the binding affinity, it was also useful to determine the DNA surface density, *Г*_DNA_, at least when the highest concentration of hCG was tested. Based on the integrated charge of surface-bound [Ru(NH_3_)_6_]^3+^, *Г*_DNA_ was calculated from Equations (8) and (9):

(8) $$\Gamma_{Ru}=Q/nFA$$

(9) $$\Gamma_{DNA}=\Gamma_{Ru}\left(z/m\right)N_{A}$$

where *n* is the number of electrons involved in the redox reaction, *F* is Faraday’s constant, *A* is the electrode area, *z* is the valence of the redox cation, *m* is the number of nucleotides in the DNA strands, and *N*_A_ is the Avogadro constant. These two equations have been proved to be applicable under the afore-mentioned assumptions [22,29]. The calculated rather low DNA surface density, (6.1 ± 1.0) × 10^11^ molecules/cm^2^, confirms that the amount of immobilized anti-hCG α antibodies was much less than the theoretical amount (<1/30) [40]. In fact this might have helped to minimize the steric hindrance among the rather large antibodies. As a result, we confirmed that the binding between anti-hCG α antibodies and hCG is as strong as that in solution.

### 3.2. Selectivity and Performance Optimization

Having shown the sensitivity enhancement of the hCG immunoassay by DNA-redox cation interaction, the next task is to confirm that other hormones do not hinder its performance. Two other hormones, follicle stimulating hormone (FSH) and thyroid stimulating hormone (TSH) were chosen for their structural similarities to hCG; the α subunit of hCG is identical to that of FSH and TSH while the β subunit is unique to hCG. The normal range of TSH in the human body is 0.4–4 μIU/mL while the normal FSH level depends on a person’s age and gender: for male subjects, the normal range is 0 to 12.4 mIU/mL and for female subjects still menstruating it is 4.7–21.5 mIU/mL. The concentrations of TSH and FSH were chosen to be at least five times higher than their normal values in the selectivity tests. 

**Figure 4:**
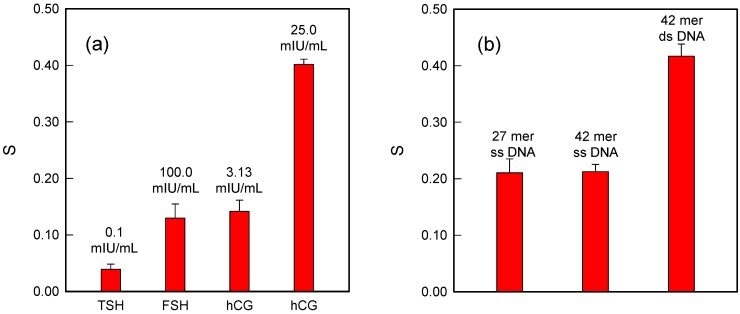
(**a**) Comparison of the sensor signal of hCG and the other two hormones (FSH, TSH) when tested with the sandwich-format immunoassay; (**b**) Signal comparison for the sandwich-format immunoassay with DNAs of different lengths. From left to right: 27-mer single-stranded DNAs, 42-mer single-stranded DNAs, and 42-mer double-stranded DNAs.

Figure 4a shows that 0.1 mIU/mL TSH barely influenced the hCG detecting system while 100 mIU/mL FSH slightly affected the performance of this sandwich-format immunosensor. However, this influence would be insignificant if such a high concentration of FSH were applied in real-world tests. Considering that FSH is an acidic protein in the human body with a pI value between 3.3 and 5.8; under the experimental conditions, FSH would be negatively charged and bind redox cations ([Ru(NH_3_)_6_]^3+^), resulting in a rather high background compared to TSH [41].

To further verify the principle of DNA-redox cation interactions for signal enhancement, the sandwich-format immunoassay was constructed with DNA strands of different lengths. Figure 4b shows that the signal from 27-mer single-stranded DNAs was not much different from that obtained from 42-mer single-stranded DNAs. In contrast, the 42-mer double-stranded DNAs produced a much higher signal, which was almost twice as strong as the signal from the 42-mer single-stranded DNAs. 

The 42-mer single-stranded DNAs showed only a slightly higher signal than the 27-mer single-stranded DNAs, which is rather surprising. This could be the result of steric hindrance by the longer DNA chain. It may be easier for the 27-mer DNA strands to form a DNA layer without interfering with other strands, and the electrostatic repulsions may be more pronounced for the 42-mer ones. Considering all the possibilities, it is reasonable to conclude that the 42-mer DNA strand does not help to improve the sensor signal (although in principle it should produce a 1.5 times higher signal than the 27-mer strand). As for the double-stranded DNAs, the results indicate a nicely enhanced signal compared to the single-stranded DNAs. This could be due to the higher negative charge density of the double helices whose phosphate backbones are more extended to the solution. Therefore, the electrostatic interaction between them and [Ru(NH_3_)_6_]^3+^ cations is much stronger than that of the single-stranded DNAs. 

Using double-stranded DNA for signal enhancement may lead to additional difficulties for practical applications. As a hybridization step is needed for the formation of double stranded DNA, longer assay times as well as more stringent experimental conditions are required. Due to the fact that the use of 27-mer single stranded DNA meets the needs of simplicity while it still produces satisfactory results, all the following experiments were carried out with 27-mer single-stranded DNA. The ideal DNA strand for maximum signal deserves further studies with strands of different lengths and sequences. 

### 3.3. Real Sample Testing and Validation

**Figure 5:**
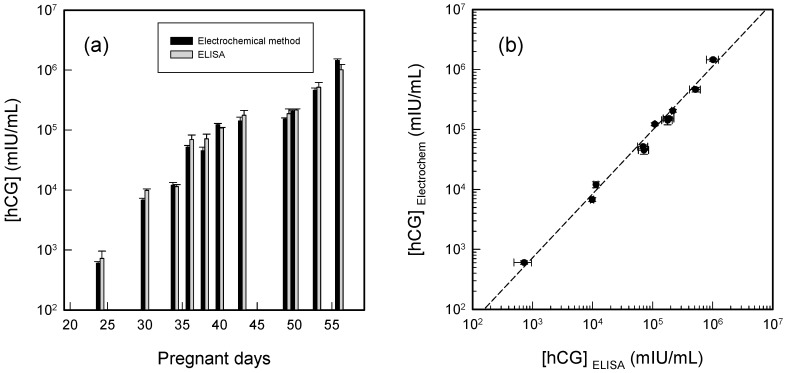
(**a**) Quantitation of the urine hCG level of a pregnant woman after various pregnancy days as measured by two different methods (electrochemical immunoassay and ELISA). The experimentally determined hCG concentrations have been multiplied by the dilution factors to reveal the actual hCG changes in urine samples; (**b**) Correlation between the hCG concentrations determined by the electrochemical method and by ELISA from the data shown in Figure 5a. The dash line indicates the best linear fit with *R*^2^ = 0.99.

All afore-mentioned tests have been carried out in standard buffer solutions despite the fact that real-world samples are usually containing impurities and many interfering species. In particular, a patient’s urine samples are typically tested for hCG quantification. In order to prove the applicability to real-world samples, urine samples from a pregnant woman from the 24th to the 57th gestation day have been tested with the sandwich-format electrochemical immunoassay. The urine samples have been diluted in order to fit in the linear response range of the electrochemical immunosensor. For a clear illustration of hCG concentration changes for the pregnant woman in her urine sample, the experimentally determined hCG concentrations have been multiplied by the dilution factors and then plotted. Subsequent comparison with the ELISA results was also performed. As shown in Figure 5a, the hCG concentration determined with the electrochemical method clearly shows an increasing trend in the early pregnancy days, and the ELISA results showed the same. It is expected that the hCG level increases exponentially after conception and implantation until the week 8 [24], which has also been confirmed previously by our DVD-based bioassay studies [25]. 

For a better understanding of the correlation between the results obtained by the electrochemical method and by ELISA, in Figure 5b we have plotted the correlation between the concentrations determined by the two methods for the same set of samples. The slope of the best fitting line was calculated to be 0.90 ± 0.03, which indicates that the hCG amounts determined by the electrochemical method are very close to those determined from the ELISA kits. The slightly lower values (indicated by non-unity slope) are probably due to the time difference of running the two sets of tests. The de-freezing processes may speed up the degradation of hCG in the urine, resulting in the observed minute difference between the two methods. The constantly lower results obtained by the electrochemical method confirm this assumption. The coefficient of determination, *R*^2^ was calculated to be 0.99, which confirms that the electrochemical method is comparable to the widely applied ELISA method from the quantitation point of view. 

As mentioned before, the hCG detection is merely a model system to prove that this DNA-redox cation interaction-based signal enhancement strategy works well. Other analytes could be tested by using the similarly designed electrochemical immunoassay with the same signal enhancement approach. It should be noted that there are still limitations of the DNA-enhanced electrochemical immunoassay method, such as the high background signal and rather narrow detection range. Further experimental improvements are warranted to overcome these limitations, which are beyond the scope of the present work (proof-of-concept).

## 4. Conclusions

Herein, a novel electrochemical method for protein detection has been developed based on a DNA-redox cation interaction enhancement strategy. Instead of labelling the antibodies with fluorophores or redox active groups, they were tethered with DNA single strands; we simply relied on the electrostatic interaction between redox cations ([Ru(NH_3_)_6_]^3+^) and negatively charged DNA backbone to obtain enhanced electrochemical signals. Human chorionic gonadotropin (hCG) detection has been studied as a model system. A response range up to 25 mIU/mL and a detection limit of 1.25 mIU/mL have been determined; these values are comparable with the results of the enzyme-linked immunosorbent assay (ELISA) for hCG quantitation. It also shows high selectivity towards hCG over other hormones such as thyroid stimulating hormone (TSH) and follicle stimulating hormone (FSH). Our approach bears the merits of cost effectiveness and simplicity of instrumentation in comparison to conventional, optical detection methods.

## References

[1] Wild D. (2005). The Immunoassay Handbook.

[2] Huels C., Muellner S., Meyer H.E., Cahill D.J. (2002). The impact of protein biochips and microarrays on the drug development process. Drug Discov. Today.

[3] Morris B.A., Clifford M.N., Jackman M.N. (1988). Immunoassays for Veterinary and Food Analysis-1.

[4] Knopp D. (2006). Immunoassay development for environmental analysis. Anal. Bioanal. Chem..

[5] Heineman W.R., Halsall H.B. (1985). Strategies for electrochemical immunoassay. Anal. Chem..

[6] Lequin R.M. (2005). Enzyme immunoassay (EIA)/enzyme-linked immunosorbent assay (ELISA). Clin. Chem..

[7] Engvall E., Perlmann P. (1971). Enzyme-linked immunosorbent assay (ELISA) quantitative assay of immunoglobulin G. Immunochemistry.

[8] Josephy P.D., Eling T., Mason R.P. (1982). The horseradish peroxidase-catalyzed oxidation of 3, 5, 3′, 5′-tetramethylbenzidine. J. Biol. Chem..

[9] Basu A., Shrivastav T.G., Maitra S.K. (2005). Development of isotopic and non-isotopic microwell based immunoassays for hCG using 125I and biotin labeled hCG. J. Immunoass. Immunochem..

[10] Hamaguchi Y., Kato K., Fukui H., Shirakawa I., Okawa S., Ishikawa E., Kobayashi K., Katunuma N. (1976). Enzyme-linked sandwich immunoassay of macromolecular antigens using the rabbit antibody-coupled glass rod as a solid phase. Eur. J. Biochem..

[11] Chen W.C.W., Nie S. (1998). Quantum dot biochnjugates for ultrasensitive nonisotopic detection. Science.

[12] Immoos C.E., Lee S.J., Grinstaff M.W. (2004). DNA-PEG-DNA triblock macromolecules for reagentless DNA detection. J. Am. Chem. Soc..

[13] Shen J., Li Y., Gu H., Xia F., Zuo X. (2014). Recent development of sandwich assay based on the nanobiotechnologies for proteins, nucleic acids, small molecules, and ions. Chem. Rev..

[14] Zhang B., Liu B., Chen G., Tang D. (2015). Redox and catalysis “all-in-one” infinite coordination polymer for electrochemical immunosensor of tumor markers. Biosens. Bioelectron..

[15] Tang D., Ren J. (2008). *In situ* amplified electrochemical immunoassay for carcinoembryonic antigen using horseradish peroxidase-encapsulated nanogold hollow microspheres as labels. Anal. Chem..

[16] Tran H.V., Piro B., Reisberg S., Nguyen L.H., Nguyen T.D., Duc H.T., Pham M.C. (2014). An electrochemical ELISA-like immunosensor for miRNAs detection based on screen-printed gold electrodes modified with reduced grapheme oxide and carbon nanotubes. Biosens. Bioelectron..

[17] Zhang J., Chen X., Yang M. (2014). Enzyme modified peptide nanowire as label for the fabrication of electrochemical immunosensor. Sens. Actuators B.

[18] Akanda M.R., Aziz M.A., Jo K., Tamilavan V., Hyun M.H., Kim S., Yang H. (2011). Optimization of phosphatase- and redox cycling-based immunosensors and its application to ultrasensitive detection of troponin I. Anal. Chem..

[19] Wang Q., Song Y., Chai Y., Pan G., Li T., Yuan Y., Yuan R. (2014). Electrochemical immunosensor for detecting the spore wall protein of Nosema biobycis based on the amplification of hemin/G-quadruplex DNAzyme concatamers functionalized Pt@Pd nanowires. Biosens. Bioelectron..

[20] Wang G., Gang X., Zhou X., Zhang G., Huang H., Zhang X., Wang L. (2013). Electrochemical immunosensor with graphene/gold nanoparticles platform and ferrocene derivatives label. Talanta.

[21] Zhu S., Zhang Q., Guo L.-H. (2008). Part-per-trillion level detection of estradiol by competitive fluorescence immunoassay using DNA/dye conjugate as antibody multiple labels. Anal. Chim. Acta.

[22] Yu H.-Z., Luo C.-Y., Sankar C.G., Sen D. (2003). Voltammetric procedure for examining DNA-modified surfaces: Quantitation, cationic binding activity, and electro-transfer kinetics. Anal. Chem..

[23] Lapthorn A.J., Harris D.C., Littlejohn A., Lustbader J.W., Canfield R.E., Machin K.J., Morgan F.J., Isaacs N.W. (1994). Crystal structure of human chorionic gonadotropin. Nature.

[24] Chard T. (1992). Pregnancy tests: A review. Hum. Reprod..

[25] Li X., Weng S., Ge B., Yao Z., Yu H.-Z. (2014). DVD technology-based molecular diagnosis platform: Quantitative pregnancy test on a disc. Lab Chip.

[26] Vartiainen J., Alfthan H., Lehtovirta P., Stenman U.-H. (2002). Elevated hCG and a high proportion of hCGβ in serum preceding the diagnosis of trophoblastic disease by seven months. BJOG.

[27] Cole L.A. (2010). Quantitative hCG Assays in Human Chorionic Gonadotropin (hCG).

[28] Bidart J.M., Birken S., Berger P., Krichevsky A. (1993). Immunochemical mapping of hCG and hCG-related molecules. Scand. J. Clin. Lab. Investig. Suppl..

[29] Cheng A.K.H., Ge B., Yu H.-Z. (2007). Aptamer-based biosensors for label-free voltammetric detection of lysozyme. Anal. Chem..

[30] Ma F., Ho C., Cheng A.K.H., Yu H.-Z. (2013). Immobilization of redox-labeled hairpin DNA aptamers on gold: Electrochemical quantitation of epithelial tumor marker mucin 1. Electrochim. Acta.

[31] Ge B., Huang Y.-C., Sen D., Yu H.-Z. (2007). Electrochemical investigation of DNA- modified surfaces: From quantitation methods to experimental conditions. J. Electroanal. Chem..

[32] Su L., Sen D., Yu H.-Z. (2006). Voltammetric study of the ion-exchange binding of non-electroactive metal cations to DNA-modified surfaces. Analyst.

[33] Pang D.-W., Abruña H.D. (2000). Interactions of benzyl viologen with surface-bound single- and double-stranded DNA. Anal. Chem..

[34] Pang D.-W., Abruña H.D. (1998). Micromethod for the investigation of the interactions between DNA and redox-active molecules. Anal. Chem..

[35] Cole L.A., Ladner D.G. (2009). Background hCG in non-pregnant individuals: Need for more sensitive point-of-care and over-the-counter pregnancy tests. Clin. Biochem..

[36] Tawa K., Kondo F., Sasakawa C., Nagae K., Nakamura Y., Nozaki A., Kaya T. (2015). Sensitive detection of a tumor marker, α-Fetoprotein, with a sandwich assay on a plasmonic chip. Anal. Chem..

[37] Rucker V.C., Havenstrite K.L., Herr A.E. (2005). Antibody microarrays for native toxin detection. Anal. Biochem..

[38] Vareiro M.M.L.M., Liu J., Knoll W., Zak K., Williams D., Jenkins A.T.A. (2005). Surface plasmon fluorescence measurement of human chorionic gonadotrophin: Role of antibody orientation in obtaining enhanced sensitivity and limit of detection. Anal. Chem..

[39] Englebienne P. (1998). Use of colloidal gold surface plasmon resonance peak shift to infer affinity constants from the interactions between protein antigens and antibodies specific for single or multiple epitopes. Analyst.

[40] Tan Y.H., Liu M., Nolting B., Go J.G., Gervay-Hague J., Liu G.-Y. (2008). A nanoengineering approach for investigation and regulation of protein immobilization. ACS Nano.

[41] Ulloa-Aquirre A., Damián-Matsumura P., Jiménez M., Zambrano E., Díaz-Sánchez V. (1992). Biological characterization of the isoforms of urinary human follicle-stimulating hormone contained in a purified commercial preparation. Hum. Reprod..

